# A Rare Case of Suspected Neuronal Intranuclear Inclusion Disease Requiring Differentiation From Neuro-Behçet’s Disease

**DOI:** 10.7759/cureus.35928

**Published:** 2023-03-09

**Authors:** Kota Sugisaki, Yohei Asakawa, Keiko Kobayashi, Mikako Ito, Masao Hori

**Affiliations:** 1 Department of Rheumatology, Japanese Red Cross Mito Hospital, Mito, JPN; 2 Department of Neurology, Japanese Red Cross Mito Hospital, Mito, JPN; 3 Department of Dermatology, Japanese Red Cross Mito Hospital, Mito, JPN; 4 Department of Pathology, Japanese Red Cross Mito Hospital, Mito, JPN

**Keywords:** hla-b51, interleukin (il)-6, cerebrospinal fluid (csf), differential diagnosis, neuro-behçet’s disease, neuronal intranuclear inclusion disease

## Abstract

A 69-year-old male patient with a long-standing history of Behçet’s disease was admitted to another hospital with minor physical injuries after a traffic accident. However, the patient was subsequently transferred to our facility because of a prolonged disorder of consciousness suspected to be related to neuro-Behçet’s disease (NBD). A thorough patient evaluation for determining the most appropriate treatment course led to a strong suspicion of neuronal intranuclear inclusion disease and predominantly ruled out NBD. This eliminated the need for unnecessary immunosuppressive intervention. Thereafter, the patient was transferred to a long-term care facility. This case highlights the importance of careful identification of pathological conditions before developing a treatment plan, regardless of the presence or absence of an underlying disease.

## Introduction

Neuronal intranuclear inclusion disease (NIID) is a slowly progressive neurodegenerative rare disorder of unknown etiology, pathologically featured by the presence of eosinophil-stained nuclear inclusions in the neurons and other somatic cells. Since its clinical manifestation is generally broad, NIID diagnosis is an arduous task to establish prior to mortality. Nevertheless, since the discovery of the utility of skin biopsy in 2011 [1], the number of reported cases of NIID has rapidly escalated. Here, we report the case of an elderly male with a chronic history of Behçet’s disease (BD) who developed new neurological symptoms. We had to rule out neuro-Behçet’s disease (NBD) to establish a suitable course of action, which led to a strong suspicion of NIID. We describe this case to underscore the significance of meticulous identification of pathological conditions for formulating a medical treatment plan.

## Case presentation

The patient was a 69-year-old male who was unemployed. At age 46, he presented with recurrent oral aphthae, genital ulcers, erythema nodosum on the lower legs, polyarticular pain, and fever and was diagnosed with BD at a different hospital. Although the details of his medical history were incomplete due to scattered medical records, he received long-term treatment with prednisolone (PSL), 10 mg/day. He was transferred to our hospital at the age of 66 due to a deteriorating relationship with his previous attending physician. At our hospital, he was treated with PSL (5 mg/day) and colchicine (0.5 mg/day), and his progress was satisfactory. He did not experience any ocular symptoms. Moreover, the patient had no history of cyclosporine use. He was a current smoker with a smoking history of 40 pack-years.

During a routine visit to our clinic in late October 2021, there were no significant changes in his usual health status. However, the police notified the patient of frequent accidents and inquired about his ability to drive in the future. The outpatient physician replied that there were no medical issues for driving restrictions. Nonetheless, on the day of the outpatient visit, at approximately 7:00 pm, the patient was involved in a self-inflicted car accident and transported to another hospital for emergency hospitalization. At the time of the accident, the patient did not have a driver's license. Chest CT tomography revealed a right rib fracture. The patient underwent conservative treatment of the injury. Following hospital admission, the patient experienced prolonged disturbance of consciousness with no discernible cause. As a result, NBD was initially suspected. Consequently, he was transferred to our hospital in early November 2021. According to his wife, the patient had no notable family medical history. His wife stated that she did not know details about his most recent condition because he had been hospitalized for a long time at a nearby hospital for an orthopedic condition.

On admission, the patient's level of consciousness was assessed using the Glasgow Coma Scale and rated 13 (E3V4M6). Currently, the patient receives enteral feeding via a nasogastric tube. When asked about the hospital to which he was transferred, the patient responded by giving the name of the previous hospital. In addition, when questioned about the details of the traffic accident, the patient responded, "I don't know," as if he talked about something else. Although the patient was capable of understanding basic instructions, such as a handshake, his response was delayed, and he often repeated instructions. The patient's vital signs were stable: body temperature, 36.7°C; blood pressure, 146/91 mmHg; heart rate, 72/min; and oxygen saturation, 98% on room air. There were no signs of meningeal irritation. The patient displayed muscle weakness in the left upper and lower extremities, with a manual muscle strength testing grade of 2. However, muscle strength in the right upper and lower extremities was well maintained. No tremor or rash was noted. Neither oral ulcers nor genital lesions were observed. Cranial nerve testing revealed pupils of 3 mm bilaterally, without difference between the right and left. The pupillary light reflex was rapid bilaterally, without deviation of the eyeballs or motor deficits. Although mild dysarthria was observed, there was no facial paralysis.

Laboratory test results on admission revealed a mild elevation of C-reactive protein (CRP), which was considered nonspecific. However, blood counts and general biochemical tests, including blood glucose, ammonia levels, and thyroid function, were unremarkable. No antinuclear, anti-DNA, or anti-aquaporin 4 antibodies were detected. Urinalysis revealed no abnormalities. No pneumonia or pleural effusion was noted on chest radiography.

We did not harbor any clinical suspicion of his illness due to the passage of more than 20 years since the onset of BD. However, we deemed it essential to eliminate the possibility of NBD, which had been identified by his previous physician. Initially, we performed a cerebrospinal fluid (CSF) analysis, which revealed normal cell counts but exhibited a slight increase in protein concentration (58 mg/dL; normal, 15-45). The elevation of interleukin 6 (IL-6) in CSF, which is deemed characteristic of NBD, was mild (6.69 pg/mL). The CSF glucose level was within the normal range and oligoclonal bands were negative. Human leukocyte antigen (HLA) testing indicated that the patient was positive for A11, A24, B61, and B67 and negative for B51, which is strongly correlated with NBD.

**Figure 1:**
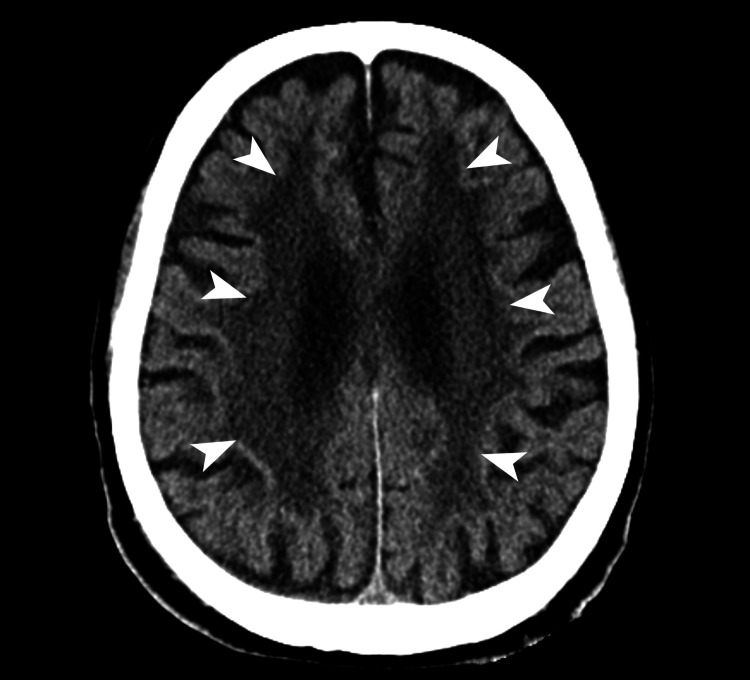
Non-contrast-enhanced head computed tomography performed in late October 2021 Atrophy of the cerebrum and diffuse low-density areas in the white matter (arrowheads) were noted.

**Figure 2:**
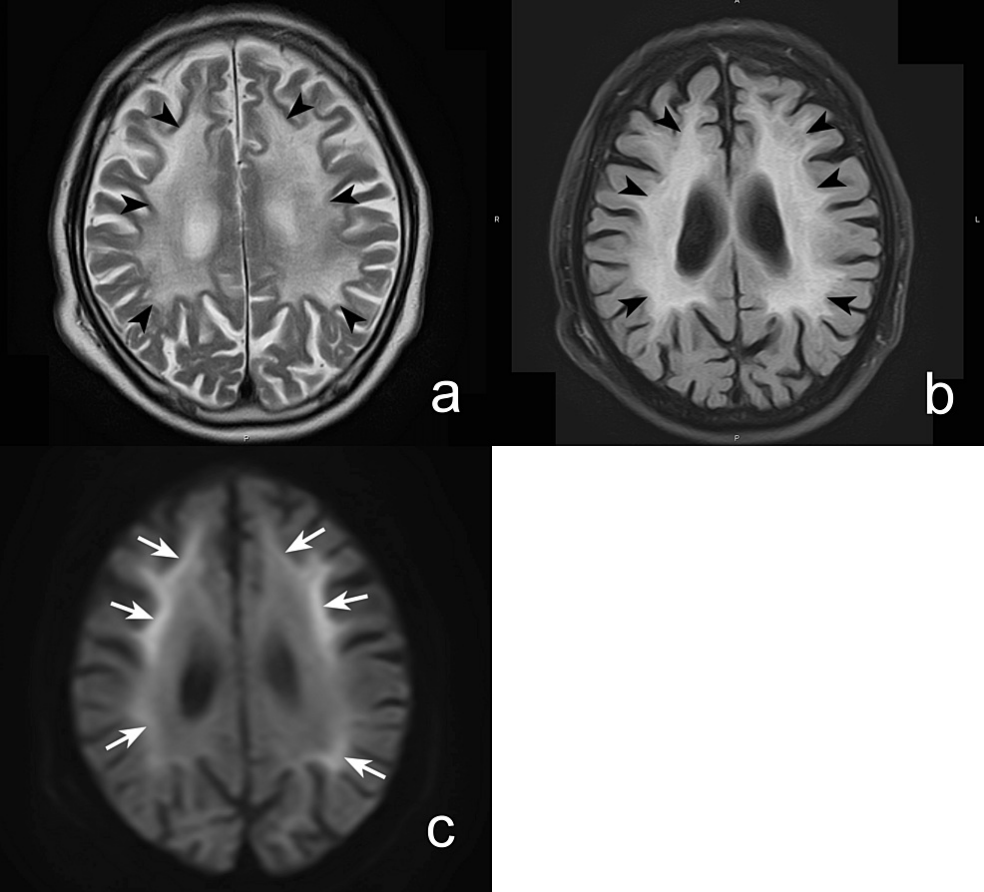
Non-contrast-enhanced head magnetic resonance imaging performed in late October 2021 Diffuse white matter lesions (arrowheads) on T2-weighted (a) and fluid-attenuated inversion recovery images (b), and characteristic high signal at the corticomedullary border (arrows) on diffusion-weighted images (c) were noted.

The previous physician had documented the presence of a symmetrical diffuse lesion in the cerebral white matter on the head CT (Figure 1) and magnetic resonance imaging (MRI) (Figure 2). However, a detailed analysis of the patient-provided images revealed a unique high signal along the cerebral cortex-medulla border on diffusion-weighted imaging (DWI), which we considered suggestive of NIID.

**Figure 3:**
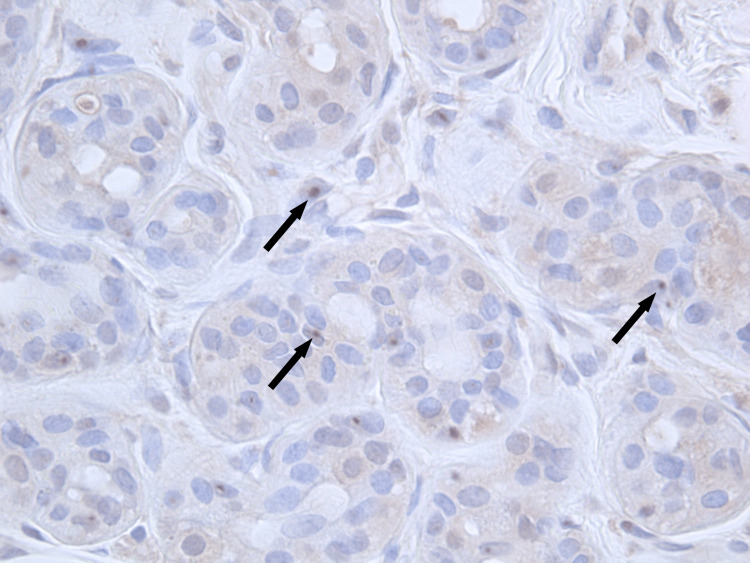
Histopathology of biopsied skin tissue collected from the abdomen in early November 2021 Anti-ubiquitin immunohistochemical staining (×400 magnification) shows multiple structures that stained positive in the nucleus of sweat glands cells (arrows).

**Figure 4:**
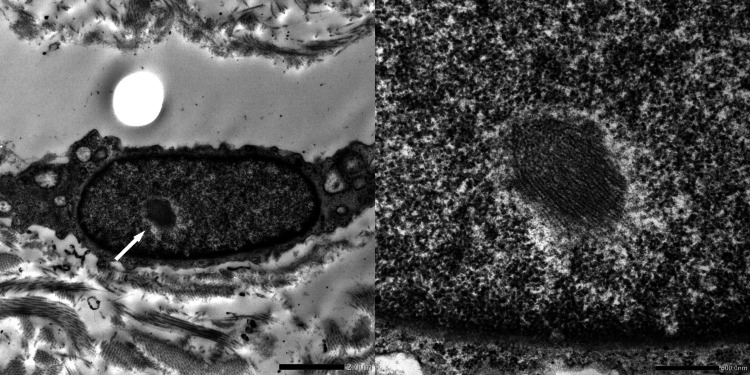
Electron microscopy Oval-shaped inclusion bodies in the nucleus of the fibrocytes were noted (arrow).

A skin biopsy from the patient's abdomen showed no anomalies using hematoxylin and eosin staining. However, immunostaining with anti-ubiquitin antibodies highlighted several structures in the cell nuclei that tested positive for inclusion bodies (Figure 3). Furthermore, electron microscopy revealed nuclear inclusions in vascular endothelial cells and fibroblasts (Figure 4). Based on these pathological findings, we strongly suspected NIID.

Although excluding fragile X-associated tremor/ataxia syndrome (FXTAS) was necessary, which could manifest similar imaging and pathological findings, genetic testing could not be performed because of the family's non-consent. Left upper and lower extremity muscle weakness necessitated a new cervical spinal cord MRI, but we could not identify the underlying cause due to the absence of specific findings.

Since we had a strong suspicion of NIID and were nearly able to eliminate NBD diagnosis, we could circumvent immunosuppressive disease-modifying therapy and instead concentrate on developing a therapeutic regimen comprising long-term care and rehabilitation. Upon admission to our hospital, the patient was already in a condition where oral ingestion was difficult, resulting in gastrostomy and continuation of enteral nutrition. The patient was eventually discharged from the hospital in late February 2022 and transferred to a recuperative facility.

## Discussion

NBD is a distinctive manifestation of BD, in addition to intestinal and vascular BD. In recent years, NBD has been subject to separate discussion, with acute NBD (ANBD) and chronic progressive NBD (CPNBD) being the two main subtypes [2]. Patients with ANBD exhibit meningoencephalitis-like symptoms accompanied by fever. ANBD is known to be induced by cyclosporine administration, which is used to treat ocular symptoms. In contrast, CPNBD is characterized by slowly progressive dementia and somatoform ataxia, which could lead to personality deterioration and immobility. Clinically, CPNBD manifests more difficulties compared with ANBD, which responds well to steroid treatment and may spontaneously resolve. CPNBD is less responsive to steroids or immunosuppressive drugs, such as cyclophosphamide, and is more likely to manifest severe psychiatric symptoms with poor prognosis after a chronic course. According to the Japanese guidelines, methotrexate (MTX) is the preferred initial treatment of CPNBD [3].

On the other hand, NIID is a neurological disease that is defined by the emergence of eosinophilic intranuclear inclusions in neurons and other organs that are positive for anti-ubiquitin antibodies. The adult form of the disease is characterized by memory impairment, disorientation, and abnormal behavior. Moreover, NIID is frequently detected in patients in their 60s to 70s [4], as in the present case. In addition to diffuse leukoencephalopathy on MRI, distinctive anomalous high signals along the corticomedullary border on DWI were observed, which prompted the diagnosis. Nuclear inclusion bodies occur not only in neurons but also in cells of various organs throughout the body, for which the diagnostic value of skin biopsy has been recognized. Genetic testing is used to differentiate NIID from FXTAS, which may present with similar imaging and pathology.

We strongly suspected NIID as the cause of the patient's condition, particularly after further diagnostic workup to exclude CPNBD. First, the period between BD onset and appearance of NBD symptoms is usually approximately five years [2], which is not consistent with the clinical course in this case. Next, based on the CSF findings, differentiation between NIID and NBD was difficult, as the elevated protein level found in this case was observed in both cases. On the other hand, elevated CSF IL-6 in NBD cases is considered to be of diagnostic value, and is known to be significantly elevated in both ANBD and CPNBD [5]. The Japanese diagnostic criteria define an increase of 17.0 pg/mL or more as a diagnostic value for CPNBD [3]. The CSF IL-6 elevation in this case was minor and not considered suggestive of NBD. Furthermore, it is known that the HLA-B51 positivity rate in CPNBD cases is much higher than that in BD cases in general, at approximately 90% [6]. However, the present case was negative for HLA-B51. Based on these negative findings, NBD was ruled out. Although genetic testing could not be performed to rule out FXTAS, we did not observe tremors or parkinsonism, which are frequently seen during the course of FXTAS. Additionally, our patient's clinical condition seemed more similar to NIID, in which cognitive dysfunction is more prominent.

Although hemiplegia is common in patients with NBD [7], in this case, NIID was more strongly suspected than NBD, as described previously. However, there have been only a few reports of NIID cases associated with hemiplegia [8,9], which is not a typical symptom of NIID. As far as the patient's family knew, the patient was able to perform daily shopping tasks without any assistance before the traffic accident. Therefore, we suggested that the weakness observed in the patient's left upper and lower limb muscles might be caused by diffuse axonal injury or other factors that could not be excluded as being related to the accident.

Currently, no targeted therapy is available for the treatment of NIID and FXTAS. In a case report of a patient with NIID and lupus nephritis-like mesangial proliferative glomerulonephritis with immunoglobulin and complement deposition, PSL (20 mg/day) was administered without improvements in proteinuria and cognitive decline [10]. One report documented the use of steroid pulse therapy for ataxia in patients with NIID and demonstrated short-term improvement [11]. However, immunosuppressive therapy for NIID and FXTAS remains experimental and there is insufficient evidence to confirm its efficacy. Conversely, for NBD, the Japanese guidelines advocate the use of PSL at moderate to high doses, including steroid pulse therapy for ANBD and MTX or infliximab for CPNBD [2].

In this case, the exclusion of NBD obviated the need for immunosuppressive treatment. When a new neurological symptom arises in a patient with a pre-existing disease, it is natural to initially consider a link to the underlying pathology. However, if diagnosis leads to a significant alteration in the treatment plan, such as an escalation of immunosuppression, a more cautious evaluation is required in relation to the underlying disease.

## Conclusions

We encountered an older man who presented with persistent disturbance of consciousness following a traffic accident. Initially, NBD was suspected due to his medical history of BD. However, after thorough examination, it was almost ruled out, and NIID was highly suspected. When a patient with a pre-existing condition develops new neurological symptoms, the underlying disease is often the primary cause of concern. However, it is crucial to conduct meticulous differential diagnoses to establish an appropriate treatment strategy.
